# Benchmarking taxonomic classifiers with Illumina and Nanopore sequence data for clinical metagenomic diagnostic applications

**DOI:** 10.1099/mgen.0.000886

**Published:** 2022-10-21

**Authors:** Kumeren N. Govender, David W. Eyre

**Affiliations:** ^1^​ Nuffield Department of Medicine, John Radcliffe Hospital, University of Oxford, Oxford, UK; ^2^​ Big Data Institute, Nuffield Department of Population Health, University of Oxford, Oxford, UK

**Keywords:** diagnosis, infection, metagenomics, simulation, taxonomic classification, whole-genome sequencing

## Abstract

Culture-independent metagenomic detection of microbial species has the potential to provide rapid and precise real-time diagnostic results. However, it is potentially limited by sequencing and taxonomic classification errors. We use simulated and real-world data to benchmark rates of species misclassification using 100 reference genomes for each of the ten common bloodstream pathogens and six frequent blood-culture contaminants (*n*=1568, only 68 genomes were available for *Micrococcus luteus *). Simulating both with and without sequencing error for both the Illumina and Oxford Nanopore platforms, we evaluated commonly used classification tools including Kraken2, Bracken and Centrifuge, utilizing mini (8 GB) and standard (30–50 GB) databases. Bracken with the standard database performed best, the median percentage of reads across both sequencing platforms identified correctly to the species level was 97.8% (IQR 92.7:99.0) [range 5:100]. For Kraken2 with a mini database, a commonly used combination, median species-level identification was 86.4% (IQR 50.5:93.7) [range 4.3:100]. Classification performance varied by species, with *Escherichia coli* being more challenging to classify correctly (probability of reads being assigned to the correct species: 56.1–96.0%, varying by tool used). Human read misclassification was negligible. By filtering out shorter Nanopore reads we found performance similar or superior to Illumina sequencing, despite higher sequencing error rates. Misclassification was more common when the misclassified species had a higher average nucleotide identity to the true species. Our findings highlight taxonomic misclassification of sequencing data occurs and varies by sequencing and analysis workflow. To account for ‘bioinformatic contamination’ we present a contamination catalogue that can be used in metagenomic pipelines to ensure accurate results that can support clinical decision making.

## Data Summary

Simulated data were produced as described and real-world data were used from previous published works [NCBI project accession numbers PRJNA690682, PRJNA604975]. We confirm all supporting data, snippets of code and protocols have been provided within the article or through supplementary data files in order for these results to be replicable.

**Impact Statement**

Metagenomics may transform clinical microbiology by enabling more rapid species detection in a potentially unbiased manner and reducing reliance on culture-based approaches. However, it is still limited by ongoing challenges such as sequencing and classification software errors. In this study, we use simulated and real-world data to define the intrinsic rates of species misclassification that occur using Illumina and Oxford Nanopore sequencing platforms with commonly used taxonomic classification tools and databases. We quantify the extent of ‘bioinformatic contamination’ arising from the classification process. This enables us to identify the best performing tools that maximize classification accuracy, and to suggest how taxonomic misclassification can be formally accounted for in clinical diagnostic workflows. Specifically, we specify thresholds for identifying or excluding polymicrobial infections in metagenomic samples, based on rates of misclassification of similar species, which might have clinical implications when treating infection.

## Background

Reliable identification of bacterial species is a fundamental part of clinical microbiology. Traditionally identification has been based on growth on selective media, morphological appearances and biochemical tests. However, many microbiology laboratories now rely largely on MALDI-TOF mass spectrometry of cultured isolates to identify most species. In clinical metagenomics, a culture-independent approach is used to identify species present in a sample based on unselective DNA sequencing.

Multiple studies have compared the potential of metagenomic approaches to replicate traditional workflows, with sensitivity for species detection, regarding culture-based approaches as the reference standard, varying from 63–98% and specificity from 35–100% [1]. Metagenomic species identification depends on the quality and properties of the sequence data used, e.g. long-read vs. short-read, the taxonomic classification algorithm and the database used to match sequences to species. Additionally approaches need to account for contamination while maximizing sensitivity and specificity. Contamination with human DNA is expected and can also arise from imperfect sampling, the laboratory environment, equipment and reagents, and also bioinformatically. Bioinformatic contamination can occur from errors in demultiplexing where multiple samples are run at once using different indexes, and from misclassification of sequence data as the wrong genus or species.

One of the primary challenges in metagenomics is reads may be generated from multiple species rather than just one following sequencing of an isolate from culture. The creates a computational challenge, i.e. how to accurately identify and differentiate all species contained within a sample, while ruling out any species present only as artefacts? This must also be done against an ever-increasing number of previous sequences to compare with. Metagenomic classifiers can be grouped based on their reference database type, which include DNA-to-DNA methods for example, Kraken [2], Kraken2 [3], Bracken [4] and PathSeq [5]; DNA-to-protein methods for example, Kaiju [6] and Diamond [7]; and DNA-to-marker methods, which include only specific gene families in their reference databases for example, MetaPhlAn [8] and mOTUs [9]. In a recent benchmarking study [10], DNA-to-DNA methods were the best-scoring methods, while DNA-to-marker methods performed more poorly.

Some studies have benchmarked classifiers and observed that the performance of different classifiers varies significantly even on the same benchmark datasets [10–15]. However, performance depends both on the taxonomic classifier and the reference database of sequences of known species used and few studies have compared performance using a uniform database [10]. For example, while Ye *et al*. previously performed classification benchmarking on short-read sequencing, previous studies have not extensively benchmarked long-read sequencing data or specifically investigated the effects of database selection or read length on classification performance. Furthermore, no catalogue of how common taxonomic misclassification is by species exists in the literature to allow studies to account for bioinformatic contamination arising from the classification process [10]. Therefore, there is still a need for thorough benchmarking to shed light on the optimal selection of bioinformatic tools for metagenomics and to account for ‘bioinformatic contamination’, which is an important and potentially underrecognized issue in the field [16].

Here we use simulated and real-world data to define the intrinsic rates of species misclassification that occur using Illumina and Oxford Nanopore sequencing platforms with commonly used classification tools and databases. We simulate reads from reference genomes, rather than simulating metagenomic samples, to create a controlled series of experiments that allow us to estimate the extent of taxonomic misclassification that occurs from sequencing a range of common pathogens and contaminants. Our findings can be applied to metagenomic samples where there is evidence of the presence at least one bacterial species. We derive species-specific thresholds for identifying if the presence of a second, third, or further additional species arises from misclassification or true presence. Therefore, our approach allows the extent of bioinformatic contamination arising from taxonomic misclassification to be accounted for in clinical diagnostic workflows and polymicrobial infections to be identified more reliably.

## Methods

### Simulated data

We generated simulated sequence data for ten common blood-stream pathogens (*Staphylococcus aureus, Streptococcus pneumoniae, Streptococcus pyogenes, Enterococcus faecalis, Pseudomonas aeruginosa, Escherichia coli, Klebsiella pneumoniae, Proteus mirabilis, Bacteroides fragilis* and *Enterobacter cloacae *) and six common blood-culture contaminants (*Staphylococcus epidermidis, Staphylococcus haemolyticus, Bacillus cereus, Cutibacterium [Propionibacterium] acnes, Streptococcus mitis* and *Micrococcus luteus *). For each species, 100 randomly selected reference genomes were downloaded where possible (on the 10 January 2021) from the NCBI RefSeq database [17], i.e. 1568 genomes in total (only 68 genomes were available for *M. luteus *). The scope of this evaluation was restricted to the identification of bacterial infections only. Although we opted to not remove genomes from this random selection that overlapped with the classification database (detailed below), we performed a sensitivity analysis excluding these genomes to ensure that contamination was not being underestimated. However, genomes very similar to those in the reference database are likely to make up some of any genomes sequenced in any given setting. Therefore, our approach, taking a random selection (including overlapping genomes) may offer more representative results.

Simulated Illumina and Oxford Nanopore reads were generated from each genome, with separate simulations with and without sequencing errors. The relevance of simulations without sequencing errors is to allow estimates of what performance might be achieved as sequencing technologies improve and potentially per base error rates are reduced in newer releases [18]. Illumina reads were simulated using ART_Illumina v2.3.7 [19] (art_illumina -i reference.fna -p -l 250 f 20 m 999 s 1 -o -nf 5 -na -ss MS -na) generating reads at a 20× coverage for each reference genome (Fig. S5) (available in the online version of this article), to simulate a realistic MiSeq throughput, which is around the minimum depth previously shown to be adequate for variant identification and antimicrobial resistance prediction [20]. Oxford Nanopore reads were simulated using NanoSim-H v1.1.0.4 [21, 22] (nanosim-h reference.fna -p ecoli_R9_1D -o -n 11000), generating reads at approximately a 20× coverage per genome with a median read length of 5921 bp (IQR 2745–11709) [range 125–120218] (Fig. S6). The default ecoli_R9_1D error profile, which mimics the R9 flow cell using 1D sequencing, and the MiSeq error profile were used for Nanopore and Illumina read simulation, respectively. No pre-processing of fastq reads was performed as this was a controlled simulated experiment.

To assess potential misclassification of human reads as bacterial taxa, we performed 100 Illumina simulations from the human genome (GRCh38 downloaded from NCBI on the 19 April 2022) using ART_Illumina and set coverage at 0.032× to approximate high human contamination in metagenomic samples (art_illumina -i GRCh38.fna -p -l 250 f 0.032 m 999 s 1 -o %s -nf 5 -na -ss MS -na).

### Taxonomic classifiers and sequence databases

Previous studies have demonstrated that Kraken-based classification tools deliver faster classification speeds and similar accuracy compared to comparator tools [10, 23]. Therefore, we chose to evaluate Kraken2 (v2.0.7) [3] and Bracken (v2.5) [4], as well as Centrifuge [24], which was written to address the high memory requirements of Kraken. Mini (8 GB) and standard (30–50 GB) uniform databases were built from archaeal, bacterial, viral and human reference genomes downloaded from NCBI RefSeq on the 10 January 2021. Databases were built according to each tool’s default setting. The mini database resembled an up-to-date version of ‘MiniKraken’ limited to 8 GB, which down-samples the standard Kraken2 database using a hash function. As Centrifuge does not allow for the restriction of size when building a database, only a standard database was built. Fastq files were classified using the default settings of Kraken2 (kraken2 --db $DBNAME sequence_data.fq --report /), Bracken (bracken -d $DBNAME -i sequence_data.fq -o /) and Centrifuge (centrifuge -k 1 x $DBNAME -f / --report-file / -S /) however, for Centrifuge we set the distinct primary assignment value to 1 (-k 1) in order to provide a comparable standard where each read is assigned to only one taxonomic level. Kraken and Bracken produced the same output, while the Centrifuge output was converted to a Kraken-style report using the command (centrifuge-kreport -x $DBNAME centrifuge.output.txt >krakenstyle.output.txt).

### Real-world data

We used a convenience sample of real-world Illumina sequencing data for benchmarking, which consisted of reads generated from sequencing of 100 pure isolates of *S. aureus, E. coli* and *K. pneumoniae* from previous published works [NCBI project accession numbers PRJNA690682, PRJNA604975] [25, 26]. Sequenced isolates originated from blood-stream infections between 15 September 2008 and 01 December 2018 in Oxfordshire, UK. Available 150 bp paired-end short-read sequences were generated using the Illumina HiSeq 4000.

### Statistical analysis

We measured classifier performance as the proportion of reads classified as the correct species or correct genus, given the known species of the reference genome or clinical isolate from which reads were simulated or obtained. The reported read abundance for each species may be referred to as either ‘raw’ calculated from the relative abundance of reads from each taxa or ‘corrected’ by accounting for genome size, such that the relative number of organisms of each taxa is estimated. We used raw abundance estimates unless the correction is automatically performed by the software as in the case of Bracken.

We used generalized linear regression (with a logit link function, binomial family and robust standard errors) to investigate associations between the proportion of reads classified as the correct species and sequencing technology, taxonomic classifier, database and species.

(R model code: m=glm(proportion_reads_correctly_classified ~sequencer+error_profile+database+classifier +species, family=binomial(link=‘logit’), data=df); Robust standard errors were estimated using the sandwich library: cov.m=vcovHC(m, type = ‘HC1’), followed by std.err=sqrt(diag(cov.m)).) We also use these models to present probabilities of reads being classified correctly for specific combinations of these covariates.

Similarly, to investigate the extent to which species misclassification was because the true and misclassified genomes were similar, we used a linear model (R code: lm(ANI ~pct_at_this_level_or_lower, data)) to investigate the relationship between the average nucleotide identity (ANI) between the true species and the misclassified species and the proportion of reads misclassified as the incorrect species. ANI values for each correct–incorrect species pair were generated by taking the median value for all pairwise comparisons between 30 genomes of each species misclassified (downloaded from NCBI RefSeq on 24 June 2021) and 50 genomes of the correct species using FastANI (fastANI --ql [QUERY_LIST] --rl [REFERENCE_LIST] -o [OUTPUT_FILE]) [27]. Analyses were performed using R (v.4.1) and R Studio (v1.4.1717) [28].

To investigate the relationship between read lengths and species classification performance, we generated Oxford Nanopore and Illumina reads of varying lengths using error and error-free profiles from 100 reference genomes of *S. aureus* and *E. coli* each, and plotted read lengths vs. the proportion of reads correctly classified by Kraken2.

## Results

### Determinants of taxonomic classification accuracy

We simulated sequence data from 1568 genomes representing ten common pathogens and six common blood-culture contaminants. We chose *S. aureus *, classified with Kraken2, using a mini (8 gb) database, with simulated Illumina reads including sequencing errors as our reference category for comparisons as this is a common pathogen and common metagenomic workflow (Tables 1 and S1). Using this combination, 89.3% (95 % CI, 88.5 %:90.9 %) of reads were classified as the correct species, and 96.7% (95.3%:97.5 %) as the correct genus. We also present performance in probabilities in Tables 2 and S2.

**Table 1.** Logistic regression model for performance of species abundance classification by sequencer, classifier, database and bacterial species. An odds ratio >1 indicates improved odds of performance while <1 indicates poorer performance relative to the reference

		Descriptive: median (IQR)	Odds ratio	95%CI	*P*-value
***Multivariable reference***	Species abundance classification (Reference: *S. aureus *, Illumina with error profile, mini database, Kraken)	89.3 (88.5:90.8)	[Reference]	–	–
***Sequencing profile***	Illumina with error profile [Reference]	92.6 (63.2:97.2)	[Reference]	–	–
	Illumina with error-free profile	92.6 (63.4:97.2)	1.00	0.96, 1.04	0.9
	Nanopore with error profile	92.5 (76.5:97.4)	1.26	1.21, 1.32	<0.001
	Nanopore with error-free profile	97.7 (92.9:99.2)	3.00	2.83, 3.18	<0.001
***Database***	Mini database	90.9 (72.2:97.7)	[Reference]	–	–
	Standard database	95.5 (81.5:98.2)	1.91	1.83, 1.98	<0.001
***Classifier***	Kraken	88.4 (58.1:96.3)	[Reference]	–	–
	Bracken	97.2 (90.5:98.9)	3.26	3.31, 3.40	<0.001
	Centrifuge	94.4 (72.2:97.3)	1.14	1.08, 1.20	<0.001
***Species***	* C. acnes *	99.5 (98.7:99.8)	5.83	5.28, 6.44	<0.001
	* E. faecalis *	98.2 (96.4:99.1)	3.45	3.21, 3.70	<0.001
	* S. pyogenes *	97.6 (95.7:99.0)	2.00	1.86, 2.15	<0.001
	* S. pneumoniae *	97.3 (95.2:98.9)	1.60	1.48, 1.72	<0.001
	* P. mirabilis *	97.2 (94.9:98.6)	2.03	1.82, 2.27	<0.001
	* S. epidermidis *	97.2 (94.7:98.4)	1.73	1.54, 1.95	<0.001
	*S.haemolyticus*	96.5 (94.2:97.8)	1.93	1.80, 2.07	<0.001
	* B. fragilis *	95.4 (91.6:97.6)	1.26	1.16, 1.37	<0.001
	* S. aureus *	95.2 (88.0:98.5)	[Reference]	–	–
	* M. luteus *	89.5 (59.5:96.3)	0.37	0.34, 0.40	<0.001
	* E. coli *	88.7 (61.2:94.2)	0.36	0.33, 0.38	<0.001
	* P. aeruginosa *	87.5 (71.4:95.0)	0.37	0.34, 0.39	<0.001
	* K. pneumoniae *	85.2 (55.0:91.5)	0.23	0.22, 0.25	<0.001
	* S. mitis *	62.3 (42.7:75.2)	0.11	0.10, 0.12	<0.001
	* B. cereus *	33.6 (14.8:51.2)	0.04	0.04, 0.05	<0.001
	* E. cloacae *	11.8 (3.3:92.5)	0.06	0.05, 0.06	<0.001

**Table 2.** Predicted abundance classification for *S. aureus* and *E. coli *. Rows are arranged from highest to lowest predicted performance

Sequencer	Error profile	Database	Classifier	Predicted performance (95 % CI)
*** S. aureus ***				
Nanopore	Error-free	Standard	Bracken	98.5 (98.4:98.6)
Nanopore	Error	Standard	Bracken	96.6 (96.3:96.8)
Illumina	Error	Standard	Bracken	95.7 (95.4:96)
Nanopore	Error	Mini	Bracken	93.7 (93.2:94.1)
Nanopore	Error-free	Mini	Centrifuge	92.5 (91.8:93)
Illumina	Error-free	Mini	Bracken	92.1 (91.6:92.7)
Nanopore	Error-free	Mini	Kraken	91.5 (90.9:92.1)
Nanopore	Error	Standard	Kraken	89.6 (88.9:90.3)
Illumina	Error	Standard	Kraken	87.3 (86.4:88.1)
Nanopore	Error	Mini	Kraken	81.9 (80.8:83)
Illumina	Error	Mini	Centrifuge	80.3 (79:81.6)
Illumina	Error	Mini	Kraken	78.2 (76.9:79.5)
Illumina	Error-free	Mini	Kraken	78.2 (76.9:79.5)
*** E. coli ***				
Nanopore	Error-free	Standard	Bracken	96 (95.7:96.2)
Nanopore	Error	Standard	Bracken	90.9 (90.4:91.4)
Illumina	Error	Standard	Bracken	88.8 (88.2:89.4)
Nanopore	Error	Mini	Bracken	84 (83.2:84.8)
Nanopore	Error-free	Mini	Centrifuge	81.3 (80.2:82.4)
Illumina	Error-free	Mini	Bracken	80.7 (79.7:81.6)
Nanopore	Error-free	Mini	Kraken	79.3 (78.3:80.3)
Nanopore	Error	Standard	Kraken	75.4 (74.3:76.5)
Illumina	Error	Standard	Kraken	70.9 (69.7:72.1)
Nanopore	Error	Mini	Kraken	61.7 (60.3:63.1)
Illumina	Error	Mini	Centrifuge	59.3 (57.6:60.9)
Illumina	Error-free	Mini	Kraken	56.1 (54.7:57.6)
Illumina	Error	Mini	Kraken	56.1 (54.7:57.5)

In a multivariable model, using Oxford Nanopore (including with sequencing errors) independently improved the odds of classifying reads correctly to the species, while reducing the odds of classifying reads to the genus level [adjusted odds ratio, aOR, vs. Illumina sequencing 1.26 (95% CI, 1.21:1.32) and 0.87 (0.83:0.90), respectively]. As expected, simulating data without sequencing errors had a similar performance for Illumina, while significantly improving performance for Oxford Nanopore, however this level of performance cannot be currently achieved in reality (Table 1).

Use of the ‘standard’, i.e. larger, database independently improved species and genus level classification [aOR vs. Mini-database 1.91 (95% CI 1.83:1.98) and 3.23 (3.09:3.37), respectively]. Compared to Kraken2, Centrifuge performed similarly for species and inferiorly for genus classification [aOR vs. Kraken2 1.14 (1.08:1.20) and 0.61 (0.58:0.64), respectively], while Bracken performed superiorly for both species and genus [aOR vs. Kraken2 3.26 (2.31:3.40) and 5.87 (5.57:6.18), respectively].

Classification performance varied by species. Some species, including *C. acnes* and *E. faecalis* had a higher adjusted probability of reads being correctly classified at the species level, compared to *S. aureus *, ranging between 92.5–99.7% across different sequencers, classifiers and databases (cf. 78.2–98.5% for *S. aureus *, Table S2) [aOR ranging between 3.45 and 5.83 (Table 1)]. Whereas other species were more challenging to classify correctly, e.g. *E. coli* with a predicted adjusted probability of reads being classified correctly ranging between 56.1 % to 96.0 % [aOR vs. *S. aureus* 0.36 (95 % CI, 0.33:0.38)]. Of note, *S. mitis, B. cereus* and *E. cloacae* had seemingly poor performance ranging between 28.2–88.0%, 13.4–74.3% and 16.6–78.8%, however, these species belong to a group or complex of bacteria, which makes genomic differentiation more complex [29–31].

### Best performing combinations

**Fig. 1.**
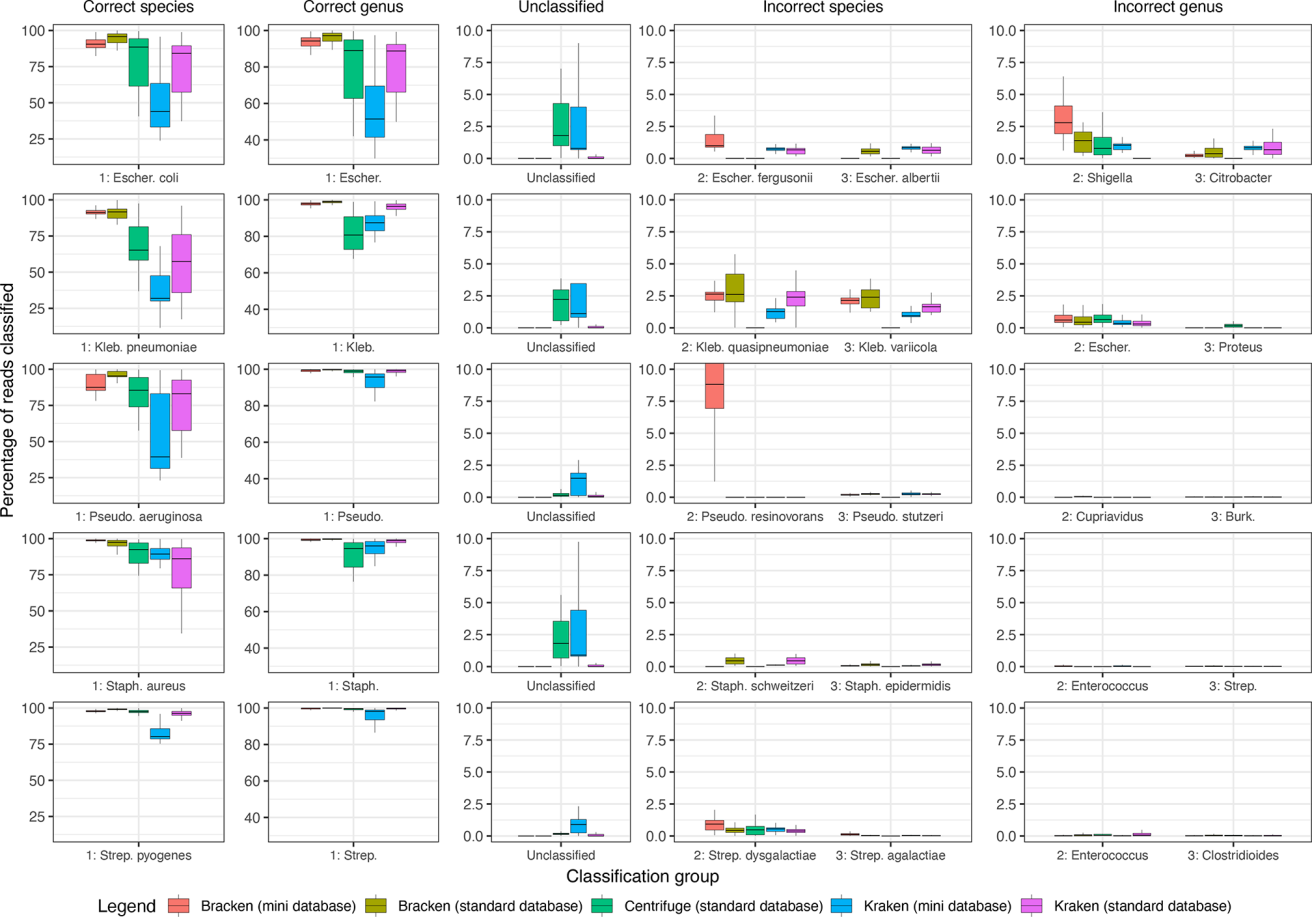
Percentage of reads classified correctly or incorrectly across classifiers for five important blood-stream pathogens. The percentage of reads with the correct and incorrect assigned species and genus, and the percentage of reads unclassified are shown.

Overall, Bracken with the standard database was the best performing option (Figs 1 and S1 show abundance classification details of five important common blood-stream pathogens and five contaminants, respectively). Across all 1568 genomes, median performance by Bracken using a standard database for species identification was 97.8% (IQR 92.7:99.0) [range 5:100] and for genus was 99.4% (IQR 98.8:99.8) [range 95.6:100.0], and had no unclassified reads. Median performance, with a common combination, Kraken2 with a mini database, for species was 86.4% (IQR 50.5:93.7) [range 4.3:100] and for genus was 94.6% (IQR 88.2:97.5) [range 76.7:100.0], with a median of 1.4% (IQR 0.8:8.9) [range 0.0:67.6] unclassified reads.

Human read misclassification was negligible. Across 100 simulations of the human genome, all classification software and database combinations performed similarly across species, genus and unclassified reads [IQR 100.0 %:100.0%; IQR 100. %:100.0% and IQR 0.0 %:0.0% respectively] except for Kraken mini database [IQR 98.6 %:98.6%; IQR 98.6 %:98.6% and IQR 0 %:0.0%, respectively] (Fig. S3).

### Choice of sequencer

**Fig. 2.**
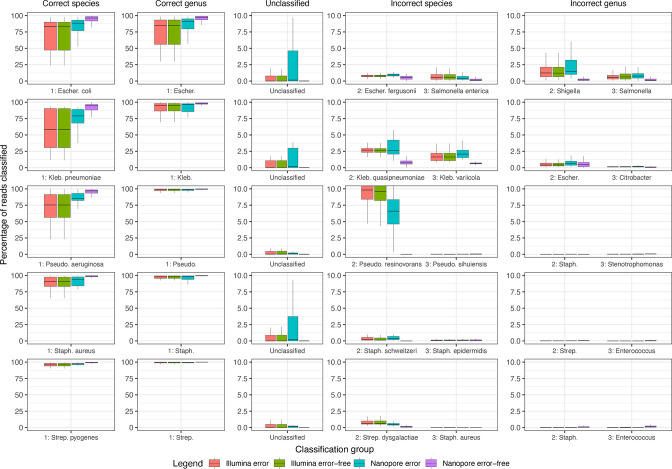
Percentage of reads classified correctly or incorrectly by sequencing device and error profile. The percentage of reads with correct and incorrect classification are shown as well as the percentage of unclassified reads.

Fig. 2 details correctly classified, unclassified and misclassified abundances for five common species according to sequencing technology and simulations with and without sequencing errors. Across all 1568 simulated genomes, the theoretical Nanopore error-free performed the best across species, genus, unclassified, misclassified species and misclassified genus abundance [IQR 95.0 %:99.3%; IQR 98.5 %:99.9%; IQR 0.0 %:0.1%; IQR 0.0 %:0.0% and IQR 0.0 %:0.4%, respectively] followed by Nanopore including simulated sequencing error [IQR 82.3 %:97.6%; IQR 92.8 %:99.3%; IQR 0 %:1.8%; IQR 0.1 %:1.9% and IQR 0 %:0.4%, respectively], Illumina error-free [IQR 77.0 %:97.6%; IQR 95.4 %:99.6%; IQR 0 %:1.2%; IQR 0.1 %:2.7% and IQR 0.0 %:0.4%, respectively] and Illumina with sequencing errors [IQR 77.0 %:97.6%; IQR 95.4 %:99.6%; IQR 0 %:1.1%; IQR 0.1 %:2.8% and IQR 0.0 %:0.4%, respectively].

### Impact of read length

**Fig. 3.**
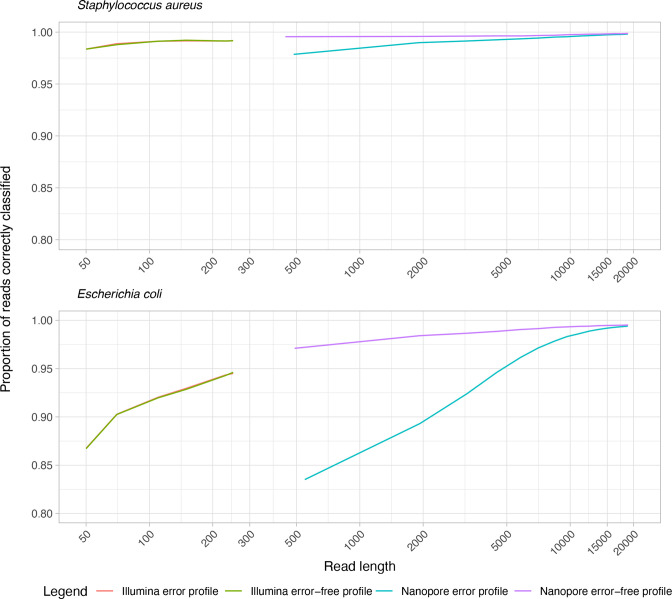
Read length vs. proportion of reads correctly classified. This figure shows the proportion of reads correctly classified against increasing read length in both Nanopore and Illumina sequencing with and without simulated errors.

Fig. 3 shows read length vs. proportion of reads correctly classified for both Illumina and Oxford Nanopore sequence simulations, with and without simulated sequencing errors. Performance for *S. aureus* and *E. coli* was similar across Illumina error (range 98.3–99.1% and 86.7–94.5%, respectively) and error-free profiles (range 98.3–99.1% and 86.7–94.6%, respectively), however, differed for Nanopore error (range 97.8–99.9% and 83.5–99.8%, respectively) and error-free profiles (range 99.5–99.9% and 97.1–99.5%, respectively). Nanopore read lengths >8 kb for *S. aureus* and >15 kb for *E. coli* performed similarly regardless of error or error-free profile. For both *E. coli* and *S. aureus *, Nanopore error profile read lengths performed similarly to Illumina error-free profile by ~2000 bp, and performed superiorly at higher read lengths >5000 bp.

### Common misclassifications

Merging data across all configurations of sequencer, classifier and database tested, the top misclassified species for *E. coli* were *Escherichia fergusonii* (median across 100 reference genomes, 0.8%; IQR 0.7 %:0.9%; 95th–99th percentile 2.5 %:3.4 %) and *Shigella flexneri* (median 0.8%; IQR 0.2 %:1.2%; 95th-99th percentile 1.5 %:1.6 %), and for genus were *Shigella* (median 1.2%; IQR 0.6 %:2.2%; 95th–99th percentile 4.8 %:5.7 %) and *Salmonella* (median 0.4%; IQR 0.2 %:0.8 %;95th–99th percentile 1.9 %:3.1 %), respectively. For *S. aureus* the top misclassified species were *S. schweitzeri* (median 0.2%; IQR 0.1 %:0.5%; 95th–99th percentile 0.9 %:1.0 %) and *P. aeruginosa* (median across 100 reference genomes, 0.0%; IQR 0.0 %:0.0%; 95th–99th percentile 2.2 %:3.6 %), and for genus were *Pseudomonas* (median 0.0%; IQR 0.0 %:0.0%; 95th–99th percentile 0.0 %:2.7 %) and *Streptococcus* (median 0.0%; IQR 0.0 %:0.0%; 95th–99th percentile 0.1 %:0.7 %), respectively.

Of note misclassified species were more common for *P. aeruginosa,* which was classified as *Pseudomonas resinovorans* (median 8.8%; IQR 6.9 %:10.7%; 95th–99th percentile 12.7 %:15.1 %), for *Streptococcus mitis* with misclassification as *S. pneumoniae* (median 9.8%; IQR 5.8 %:15.4%; 95th–99th percentile 26.6 %:31.3 %), and for *E. cloacae* were *Enterobacter hormaechei* (median 2.5%; IQR 0.9 %:54.8%; 95th–99th percentile 89.1 %:93.8 %) and *Enterobacter bugandensis* (median 0.1%; IQR 0 %:0.3%; 95th–99th percentile 89.6 %:95.6 %) were identified instead, however, these all belonged to the same species group/complex. Further detailed information on individual species and genus misclassification can be found in Table S3, while Table 3 provides a summary with recommended 95th, 99th and 100th percentile cut-off value to determine if additional organisms are present, i.e. there is polymicrobial infection.

**Table 3.** Recommended threshold abundance to call additional true species. This summary table was generated by merging data across all configurations of sequencer, classifier and database tested. Detailed threshold values for specific sequencer, classifier and database combinations can be found in Table S3

Bacteria	Correctly classified median (IQR)	Misclassified 1	95−99th percentile	Misclassified 2	95th–99th percentile	Misclassified 3	95−99th percentile	Misclassified 4	95−99th percentile
**Pathogens**									
* B. fragilis *	95.4 (91.6:97.6)	* B. ovatus *	0.7 : 1.9	* B. xylanisolvens *	1 : 1.6	* B. caecimuris *	0.3 : 0.6	*P. dorei*	0.7 : 1.4
* E. cloacae *	11.8 (3.3:92.5)	* E. hormaechei *	89.1 : 93.8	* E. bugandensis *	89.6 : 95.6	* E. roggenkampii *	7.2 : 81.6	* E. asburiae *	4.4 : 41.3
* E. faecalis *	98.2 (96.4:99.1)	* E. faecium *	0.9 : 1.9	* E. cecorum *	0.6 : 1.1	* S. haemolyticus *	0 : 3	na	na
* E. coli *	88.7 (61.2:94.2)	* E. fergusonii *	2.5 : 3.4	* S. flexneri *	1.5 : 1.6	* E. albertii *	1 : 1.2	* E. marmotae *	0.6 : 0.8
* K. pneumoniae *	85.2 (55:91.5)	* K. quasipneumoniae *	4.9 : 5.4	* K. variicola *	3.2 : 3.5	* K. aerogenes *	1.9 : 2.7	* E. coli *	1.5 : 2.6
* P. mirabilis *	97.2 (94.9:98.6)	* P. rettgeri *	1.6 : 1.8	* P. vulgaris *	0.4 : 6.9	* P. cibarius *	0.2 : 1.3	* P. terrae *	0.3 : 7.2
* P. aeruginosa *	87.5 (71.4 : 95)	* P. resinovorans *	12.7 : 15.1	* P. stutzeri *	0.4 : 0.5	* P. putida *	0.3 : 0.9	* P. fluorescens *	0.8 : 1.2
* S. aureus *	95.2 (88 : 98.5)	* S. schweitzeri *	0.9 : 1	* S. epidermidis *	0.5 : 0.8	* P. aeruginosa *	2.2 : 3.6	* S. pseudintermedius *	0 : 1.3
* S. pneumoniae *	97.3 (95.2 : 98.9)	* S. mitis *	1.8 : 2.8	* S. pseudopneumoniae *	1.4 : 2.3	* S. pyogenes *	0.1 : 0.4	*Homo sapiens*	0.1 : 1.8
* S. pyogenes *	97.6 (95.7 : 99)	* S. dysgalactiae *	1.3 : 1.9	* S. agalactiae *	0.6 : 1.1	*F. alocis*	2.3 : 2.4	* S. aureus *	0.2 : 0.7
**Contaminants**									
* B. thuringiensis *	35.2 (18.5 : 42.3)	* B. cereus *	82.8 : 95.3	* B. mycoides *	48.2 : 89.4	* B. albus *	4.4 : 9.9	*na*	na
* C. acnes *	99.5 (98.7 : 99.8)	* C. modestum *	0.4 : 0.7	* C. avidum *	0.4 : 0.8	*Homo sapiens*	0.1 : 0.3	* S. aureus *	0 : 0.7
* M. luteus *	89.5 (59.5 : 96.3)	*B. luteolum*	0.5 : 0.5	*K. sedentarius*	0.7 : 0.8	*P. sulfonivorans*	0.1 : 12	*R. kristinae*	0.6 : 1
* S. epidermidis *	97.2(94.7 : 98.4)	* S. aureus *	1.6 : 1.8	* S. hominis *	0.3 : 62.5	* S. warneri *	0.2 : 1.6	* S. pasteuri *	1.1 : 6.1
* S. haemolyticus *	96.5 (94.2 : 97.8)	* S. aureus *	2.1 : 3.8	* S. hominis *	1 : 2.1	* S. epidermidis *	1 : 1.9	na	na
* S. mitis *	62.3 (42.7 : 75.2)	* S. pneumoniae *	26.6 : 31.3	* S. oralis *	84.1 : 96.3	na	na	na	na

Of the 1568 randomly selected genomes used in simulations, 7.6% overlapped with the references genomes used to build the classification database, however, when overlapping genomes were removed from this analysis this did not affect the findings (data are not shown separately as they are very similar). A higher sequencing depth of 40× compared to 20× did not result in any improvements in classification performance or reduction in misclassified or unclassified reads (Table S4).

### Relationship between species misclassification and nucleotide identity

**Fig. 4.**
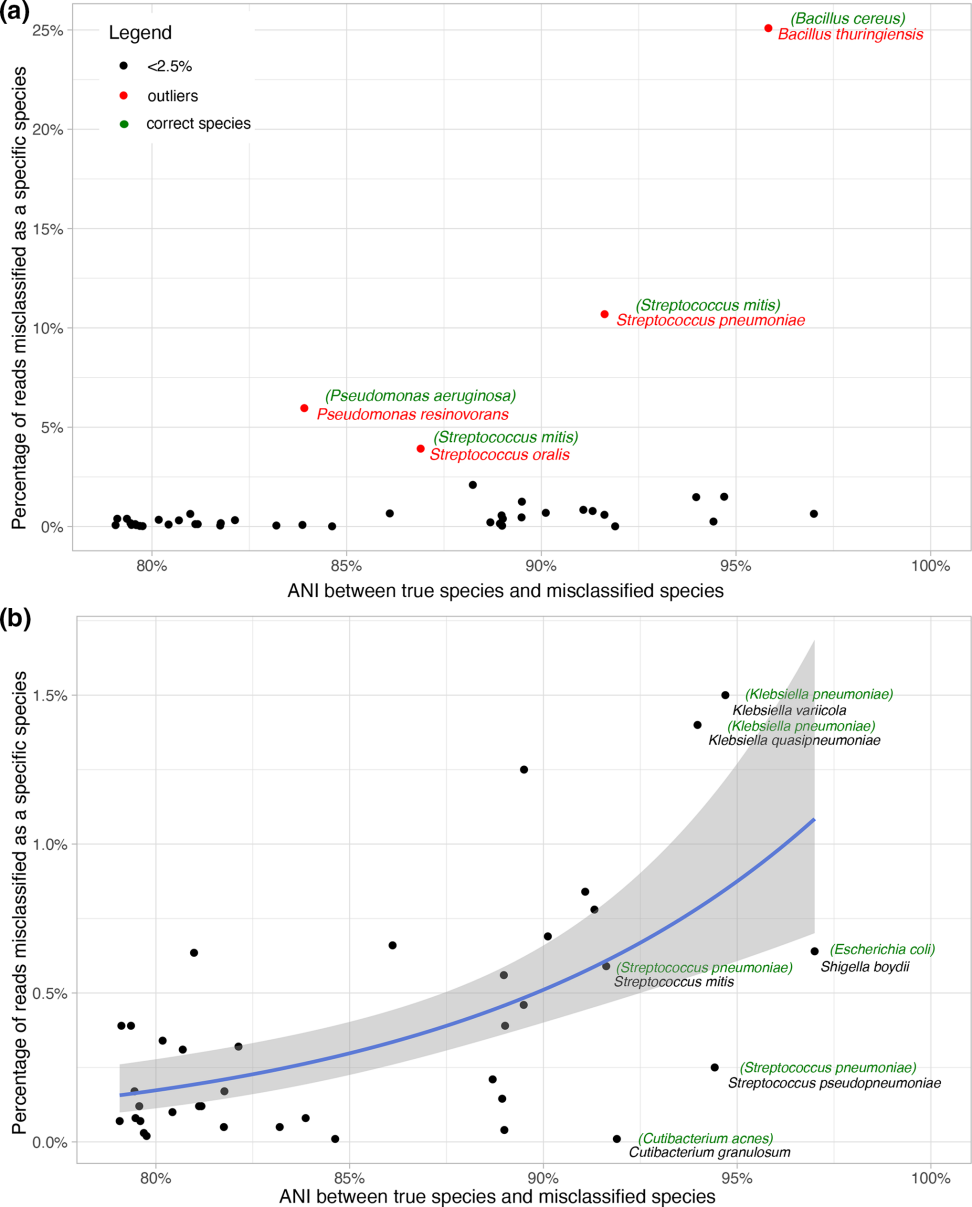
ANI between true species and misclassified species against percentage of reads misclassified as a specific species. (a) All pairs and (b) the same plot with the outliers from (a) (shown in red, with the correct species shown in green inside brackets) excluded. For (b) a generalized linear regression line (with a logit link function and binomial family) is plotted (the shaded area indicates the 95% confidence interval). The misclassified species corresponding to example outlying points are shown in black, with the corresponding correct species shown in green in brackets.

Misclassification at the species level was more common when the misclassified species had a higher average nucleotide identity to the true species (Fig. 4 and Table S5). However, there were several outliers, where misclassification was greater than expected for the given level of genome-wide nucleotide identity, for example *B. thuringiensis* was misclassified at 25% when *B. cereus* was the true species, and *S. pneumoniae* at 10% when *S. mitis* was the true species. This reflects the difficulty distinguishing species within known groups or complexes of bacteria, and potentially reflects that some parts of the genome may have higher nucleotide identity.

### Comparison with real-world data

**Fig. 5.**
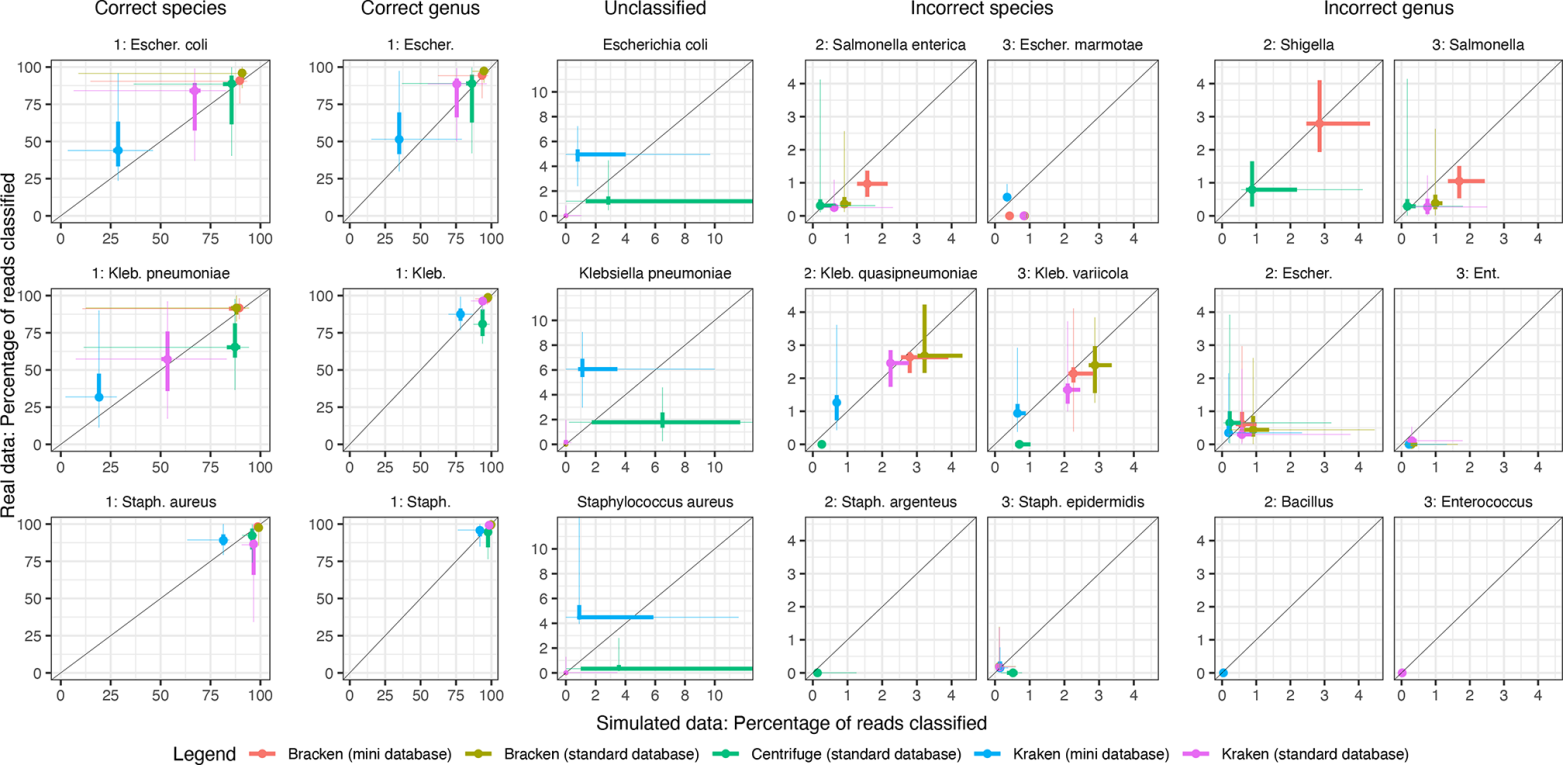
Percentage of reads classified correctly or incorrectly in Illumina simulated data and real-world data. The diagonal line indicates values with exact correspondence between the simulated and real-world data.

Findings from Illumina simulations (using MiSeq error profiles) were very similar to real-world Illumina data (generated with the HiSeq platform, Fig. 5). Real-world data from *E. coli, K. pneumoniae* and *S. aureus* performed better than simulated data both when using Kraken2 mini database (percentage of reads classified as the correct species: 46.2% vs. 28.7%; 31.9% vs. 19.1%; 89.3% vs. 81.4%, respectively) and Kraken2 standard database (84.2% vs. 67.1%; 57.4% vs. 53.5%; 86.1% vs. 96.6%, respectively). Similar bioinformatic misclassification existed in both real and simulated data. Misclassification in *K. pneumoniae* for example as *K. quasipneumoniae* was marginally higher in simulated data vs. real-world data in Bracken mini database (2.8% vs. 2.6%), Bracken standard database (3.2% vs. 2.7%) and Kraken2 standard database (2.2% vs. 2.4%).

## Discussion

The potential for metagenomic technology to improve diagnostics and patient outcomes has been widely recognized [32], and there is extensive work ongoing to overcome current challenges associated with use of these approaches in clinical settings [33]. In this article, we simulate and use real-world sequencing data to define the intrinsic rates of bioinformatic species misclassification that occur using common sequencing platforms, classification tools and databases. This enables us to quantify the extent of bioinformatic contamination in the classification process to account for this formally in diagnostic workflows, and further guide the selection of optimal bioinformatic tools to maximize classification accuracy.

Overall, across both short and long reads we found that using the Bracken classifier with a ‘standard’ database, i.e. larger resulted in the most accurate performance across all species with almost no reads unclassified (Figs 1 and S1). In comparison, Kraken2 with a mini database, a commonly used combination performed worse. Previous studies with Illumina short reads have reported estimates from Kraken2 to be more accurate than Kraken1, KrakenUniq, CLARK and Centrifuge at both the genus and species levels [3]. Furthermore, studies have shown with Illumina data that Bracken which re-assigns reads in intermediate taxonomic nodes to the species level or above from Kraken1/2 outputs produce far better abundance estimates [4, 10]. Our results confirm that Bracken produces far more accurate abundance estimates for both long- and short-reads, especially when using a standard database. Previous metagenomic studies have rarely used Bracken or standard, i.e. larger, databases [1], the latter in part because of higher memory requirements, and we encourage future studies to utilize the most accurate tools available where computational resources permit.

To formally account for bioinformatic contamination in clinical diagnostic workflows, we produced the first known catalogue of misclassified species from simulating 1568 genomes representing ten common pathogens and six common blood-culture contaminants using different classification tools, databases and sequencers (summary catalogue Table 3, and detailed catalogue Table S3). It should be noted that our proposed minimum cut-off thresholds for identifying the presence of additional species are based on the extent of bioinformatic contamination arising from taxonomic misclassification only. Other forms of contamination are possible during sampling and laboratory workflows, which also need to be considered. Our thresholds can be applied to metagenomic samples by multiplying the estimated proportion of a sample that is from a specific species by the 95, 99 or 100% misclassification cut-off values in Table 3. For instance, if a sample by read proportion is found to have 50% *Streptococcus mitis* and 40% *S. epidermidis *, then the 95% threshold for the proportion of reads misclassified as *S. pneumoniae* would be 50% * 26.6%+40% * 0%=13.3%. Therefore, even if the remaining 10% of the sample were classified as *S. pneumoniae* this would be considered a bioinformatic contaminant, while *S. mitis* and *S. epidermidis* would not as they both are above their respective modified cut-off values. Refinements to this heuristic approach are possible, for example probabilistically estimating the proportion of each sample made up of a given species while simultaneously accounting for misclassification.

We used existing real-world Illumina data to show correlation with the Illumina simulator (Fig. 5), while the Nanopore simulator used has been benchmarked extensively elsewhere [21]. Detailed 95th, 99th and 100th percentile cut-off values for other species are available in Table S3, which may be used to determine if there is strong evidence that additional organisms are present, i.e. there is polymicrobial infection or, conversely, if only bioinformatic contamination is present. Using simulations, we also show that misclassification of human reads is negligible when human DNA contamination might exist in clinical samples.

Additionally, our results show that misclassification at the species level was more common when the misclassified species had a higher average nucleotide identity to the true species. However, there were several outliers where misclassification was greater than expected for the given level of genome-wide nucleotide identity reflecting the difficulty distinguishing species within known groups or complexes of bacteria. Tools such as DeepMicrobes, which use a deep learning-based computational framework for taxonomic classification may offer an advantage in these situations where far fewer false positives are produced compared to other tools regardless of different degrees of nucleotide similarity [34].

The advent of long-read technologies such as the Oxford Nanopore has significant advantages for metagenomic analysis from the detection of structural variants to improved *de novo* assembly [35]. Furthermore, long-read sequencing of native molecules eliminate amplification bias while preserving base modifications [36]. However, these technologies are limited by a higher error-rate affecting 6–12% of all sequenced bases, with a significant fraction of insertion and deletion errors [37]. This may affect the success of current classification methods, as there are few algorithms developed to exploit long-read data. However, even after simulating sequencing errors, the longer reads of Nanopore sequencing meant that it still could outperform Illumina sequencing. This finding is supported by previous work [38]. Following on from this observation we directly investigated the impact of read length on the proportion of reads correctly classified. Allowing for sequencing error, for *E. coli* and *S. aureus *, Nanopore reads of ~2000 bp performed similarly to Illumina reads and performed better at higher read lengths >5000 bp. Our findings have several implications: as technology and accuracy for long-read sequencing advances, we expect classification accuracy to significantly improve. Furthermore, filtering shorter reads (at least <1000 bp) from error-prone Nanopore sequencing can provide performance either equivalent or superior to standard Illumina sequencing.

Although we have thoroughly analysed and discussed technical factors that correlate with taxonomic misclassification, biological factors that might be responsible for misclassification might also exist. One such biological aspect that we have addressed here is the presence of the complexes or species, which are closely related yet named differently, which can result in the erroneous classification of sequence reads. Other biological factors might include the contribution of high similar genomic regions that are not specific to a particular species but shared across two or more species. These regions are well known to be prevalent in bacterial pathogens (e.g. in mobile genetic elements) and will affect read misclassification rates even if the sequencing reads are error-free. Future studies are required that ascertain how much bioinformatic contamination may be removed by building classifier databases from modified genome-sequence databases, which contain exclusively the relatively ‘stable’ genomic backbone regions after excluding regions shared among species.

In conclusion, our findings highlight the pertinent issue of the presence of bioinformatic contamination and how this varies by the combination of sequencer, classifier and database used. While we have recommended Bracken using a standard database to be utilized in metagenomic workflows, we have produced a misclassification catalogue whereby misclassification can be accounted for using any combination of sequencers, classifiers or databases benchmarked in this study. Benchmarking metrics should inform the choice and implementation of metagenomic sequencing tools for both research and clinical applications.

## Supplementary Data

Supplementary material 1

Supplementary material 2
