# Supplementary material for: Benchmarking taxonomic classifiers with Illumina and Nanopore sequence data for clinical metagenomic diagnostic applications
Source: Microb Genom. 2022 Oct 21;8(10):mgen000886. doi: 10.1099/mgen.0.000886 (PMC9676057; doi:10.1099/mgen.0.000886)
Supplement: Supplementary material 1 [file mgen-8-886-s001.pdf]

Benchmarking taxonomic classifiers with Illumina and Nanopore sequence data for clinical metagenomic diagnostic applications: Supplementary material

Supplementary table legends

Table S1. Logistic regression model for performance of genus abundance classification by sequencer, classifier, database and bacterial species.

Table S2. Predicted abundance classification for all included species.

Table S3. Detailed threshold abundance recommended to call additional true species and genus.

Table S4. Reference list of genomes randomly selected from NCBI to perform simulation

Table S5. ANI between true species and misclassified species against percentage of reads misclassified as a specific species

Supplementary Figures

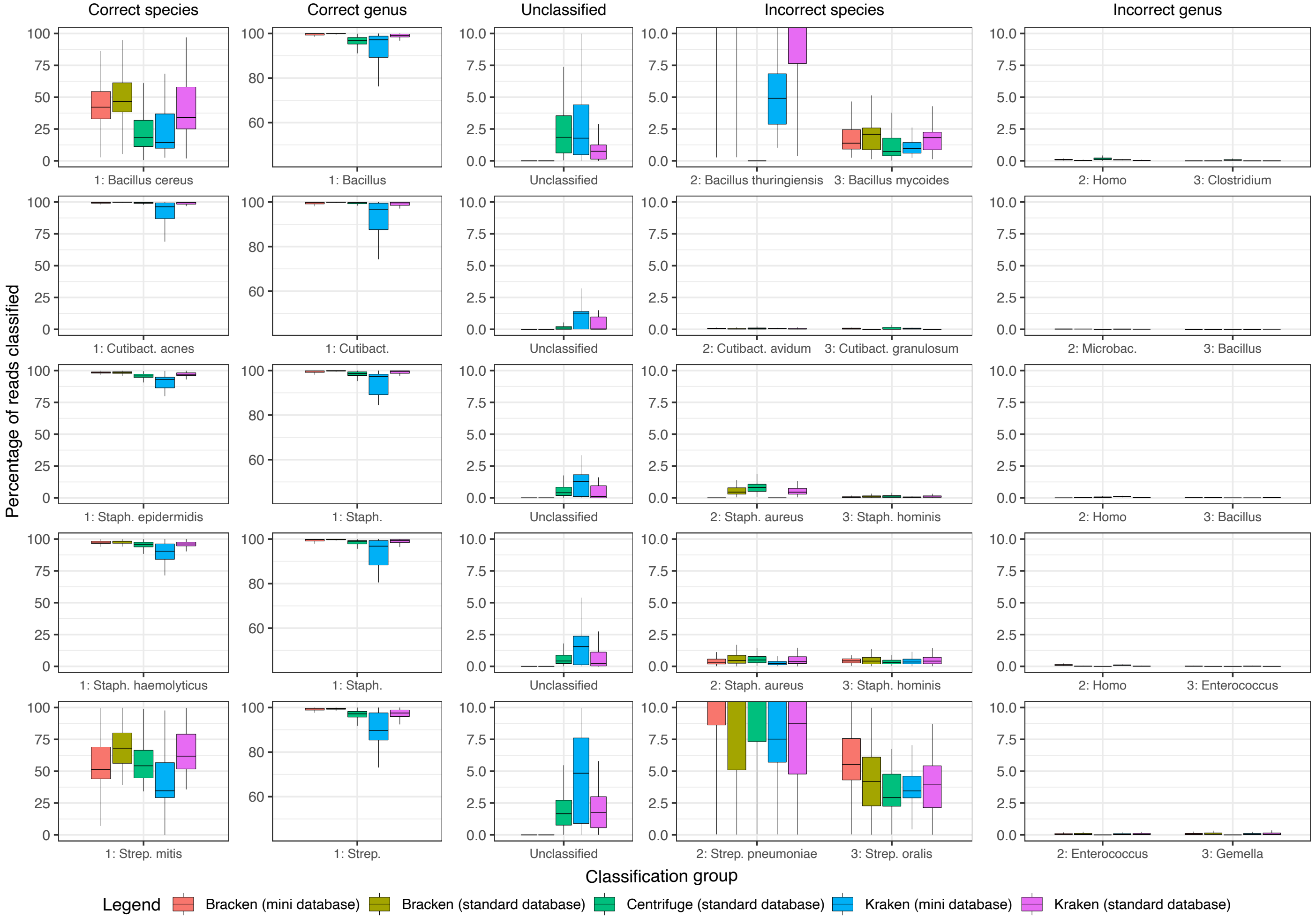

Figure S1. Percentage of reads classified across classifiers for five common blood stream contaminants. The percentage of reads with the correct and incorrect assigned species and genus, and the percentage of reads unclassified are shown.

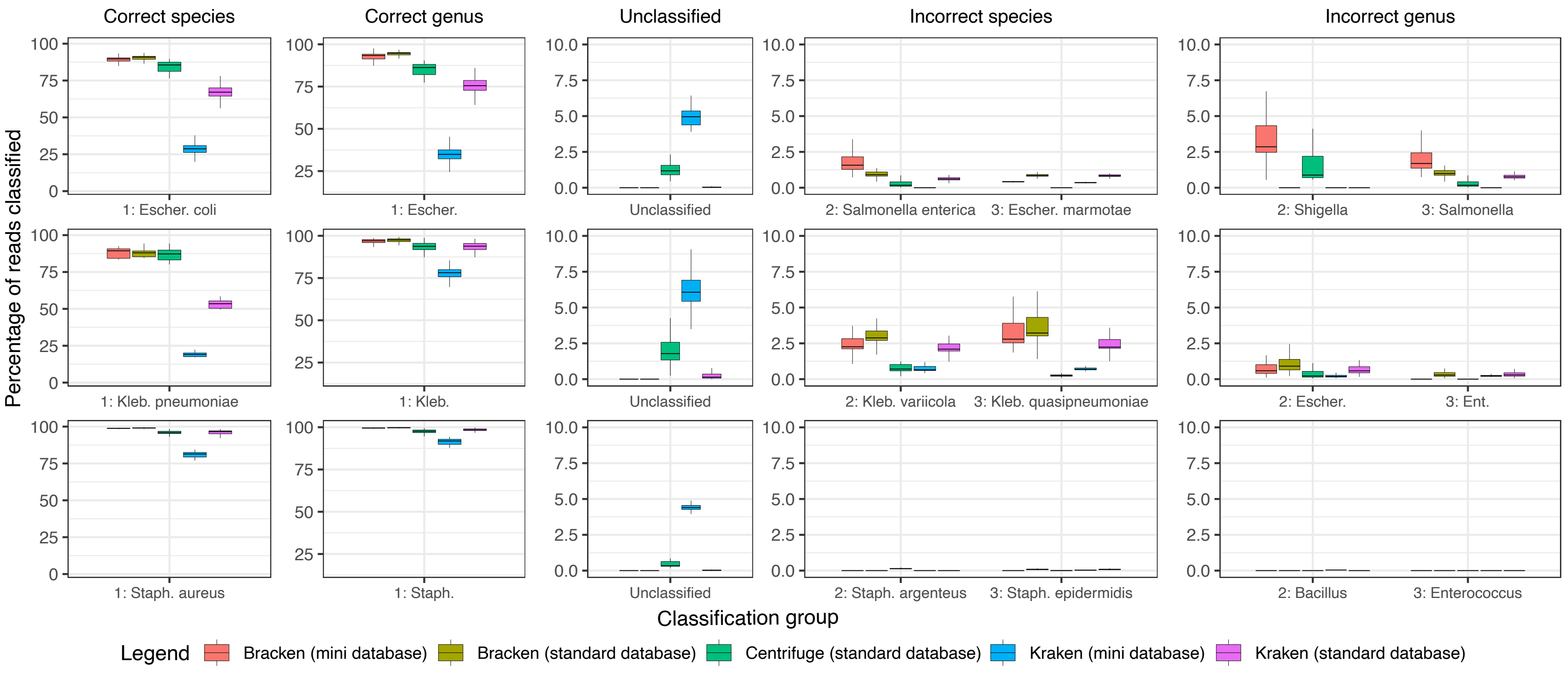

Figure S2. Percentage of reads classified of real-world pure isolates from Illumina sequencing. The percentage of reads with the correct and incorrect assigned species and genus, and the percentage of reads unclassified are shown.

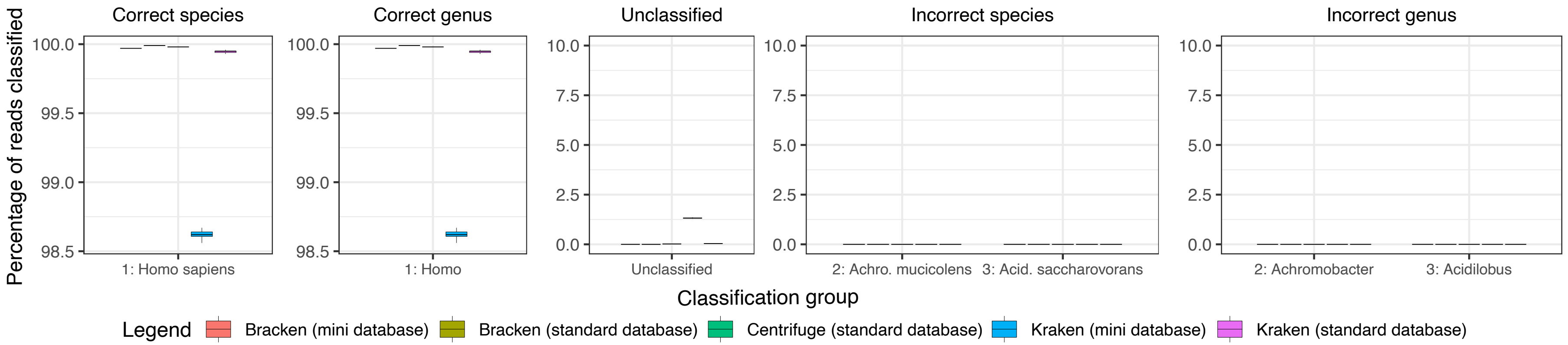

Figure S3. Percentage of reads classified from simulated human reads using Illumina sequencing. The percentage of reads with the correct and incorrect assigned species and genus, and the percentage of reads unclassified are shown.

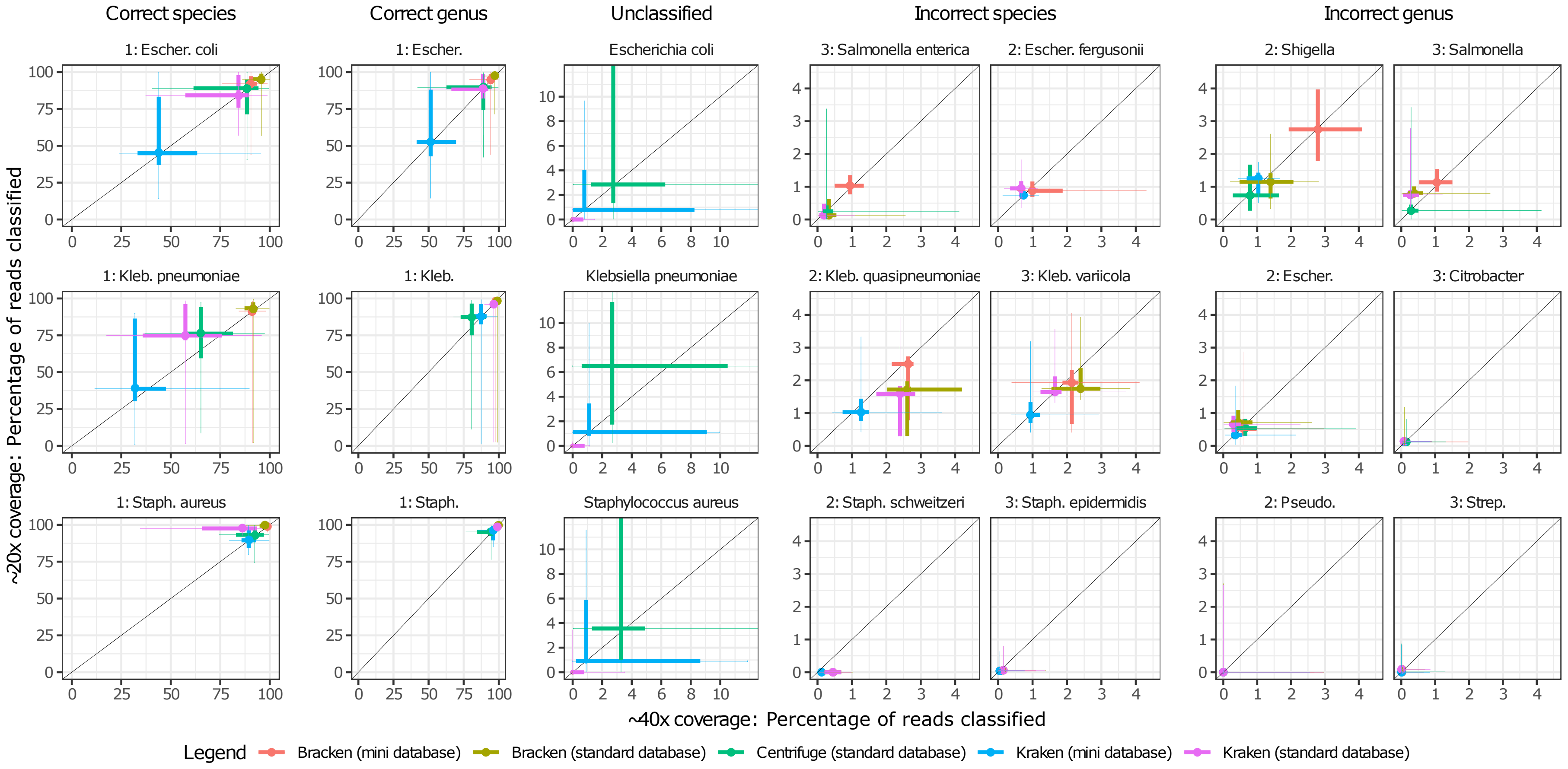

Figure S4. Percentage of reads classified at 20x vs. 40x coverage. The diagonal line indicates values with exact correspondence between 20x and 40x coverage data.

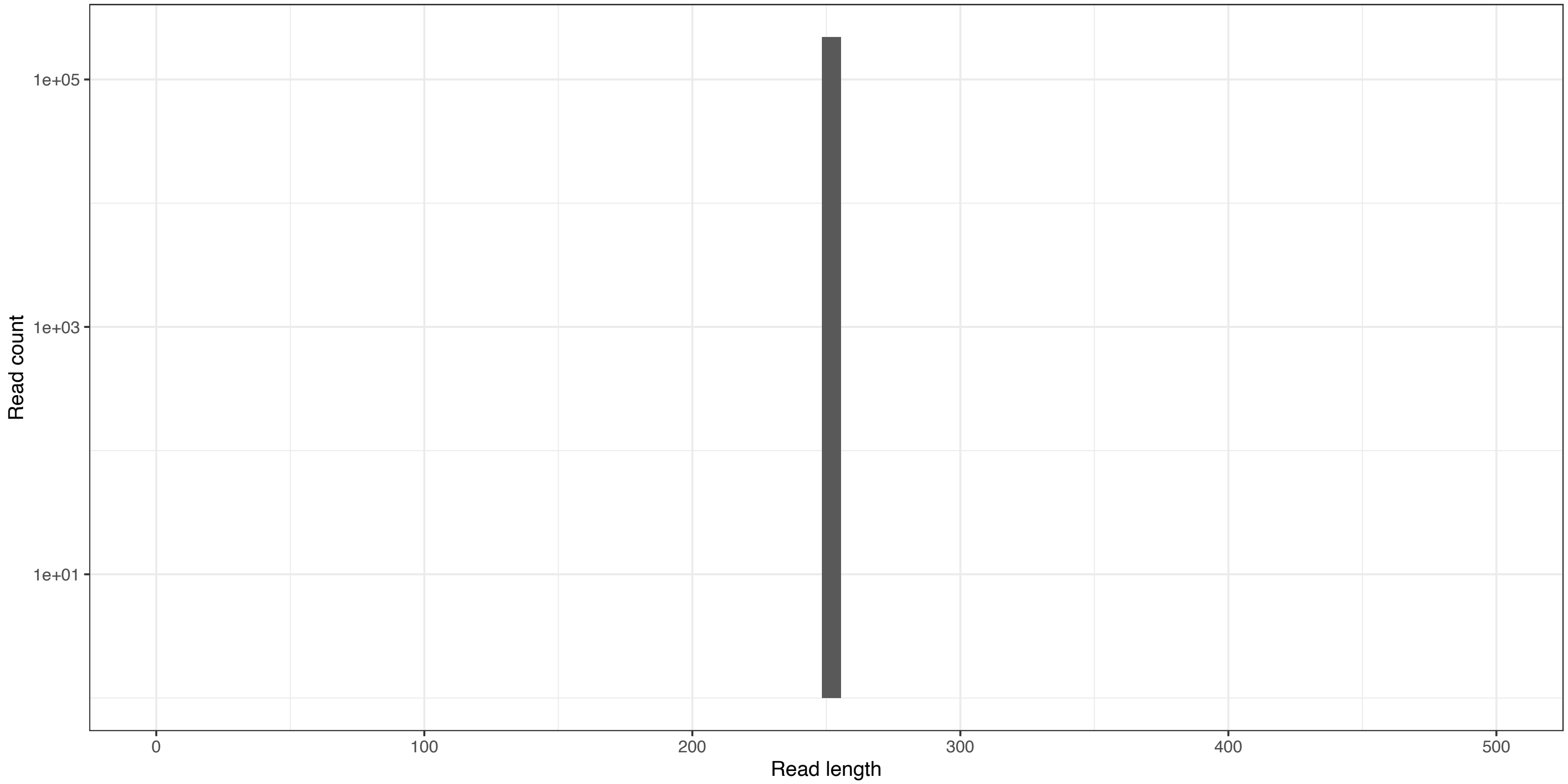

Figure S5. Illumina sequencing read distribution.

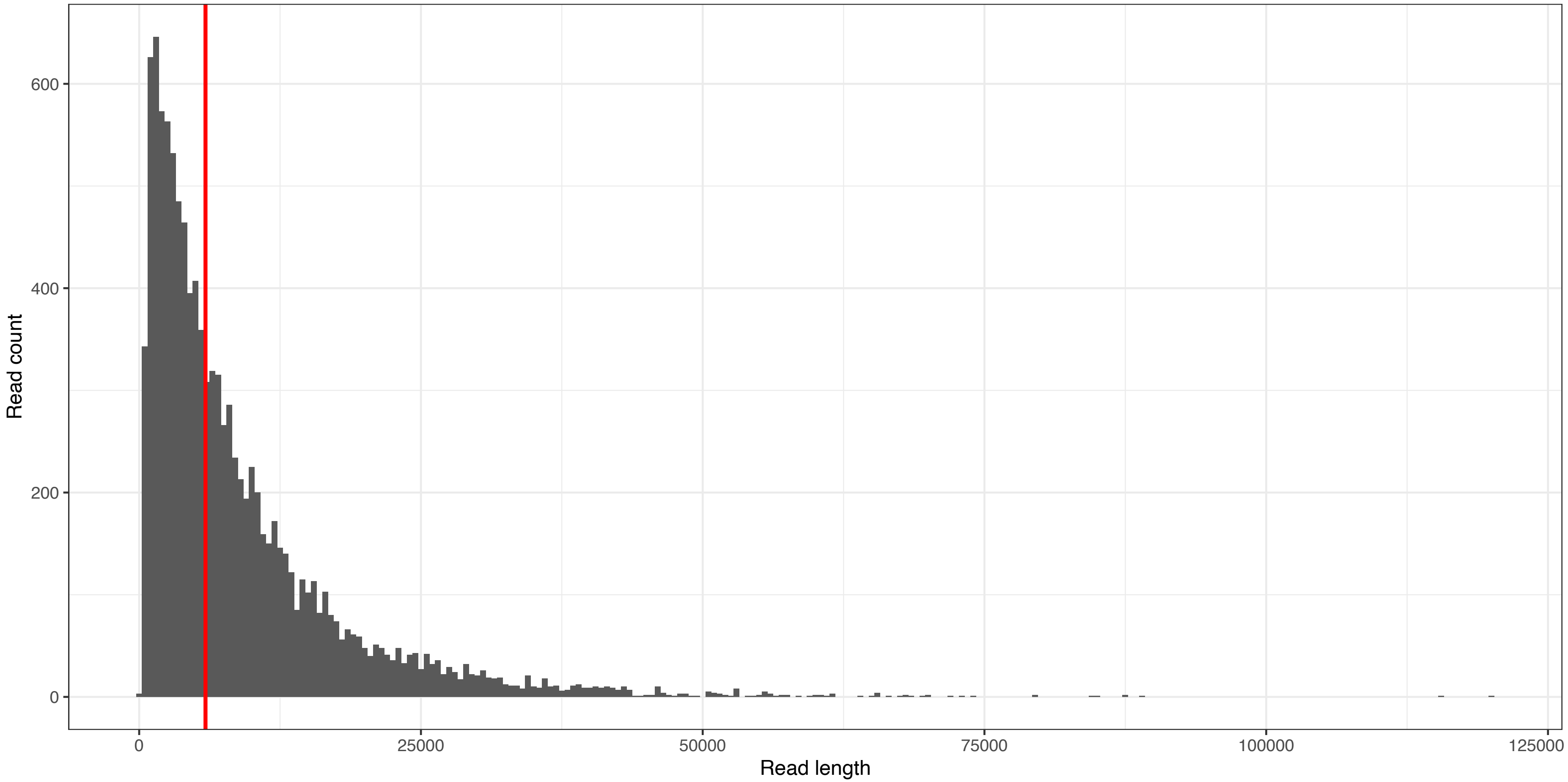

Figure S6. Nanopore sequencing read distribution.
